# Supplementary material for: Validation of automatically measured T1 map cortico-medullary difference (ΔT1) for eGFR and fibrosis assessment in allograft kidneys
Source: PLoS One. 2023 Feb 15;18(2):e0277277. doi: 10.1371/journal.pone.0277277 (PMC9931131; doi:10.1371/journal.pone.0277277)
Supplement: S1 File — (DOCX) [file pone.0277277.s001.docx]

| **Acronyms/Abbreviations** | |
| --- | --- |
| ΔADC | Cortico-Medullary Difference Of ADC |
| ∆T1 | Cortico-Medullary Difference |
| 2D U-Net | Two-Dimensional U-Net |
| 2D-CNN | Two Dimensional CNN |
| ACC | Accuracy |
| ADC | Apparent Diffusion Coefficient |
| ADPKD | Autosomal‑Dominant Polycystic Kidney Disease |
| AI | Artificial Intelligence |
| BA plots | Bland-Altman Plots |
| CKD | Chronic Kidney Disease |
| CNNs | Convolutional Neural Networks |
| CT | Computed Tomography |
| DC | Dice Coefficient |
| DCE-MRI | Dynamic Contrast Enhanced MRI |
| DL | Deep Learning |
| DWI | Diffusion Weighted Imaging |
| eGFR | Estimated Glomerular Filtration Rate |
| GPU | Graphics Processing Unit |
| HCs | Healthy Controls |
| IF | Interstitial Fibrosis |
| MAE | Mean Absolute Error |
| ML | Machine Learning |
| MRI | Magnetic Resonance Imaging |
| RAM | Random-Access Memory |
| RCM U-Net | Renal Cortex and Medulla U-Net |
| ReLU | Rectified Linear Unit |
| ResNet34 | A Residual Network With 34 Convolutional Layers |
| ResNet50 | A Residual Network With 50 Convolutional Layers |
| RMSProp | Root Mean Squared Propagation |
| ROIs | Region of Interests |
| SE | Sensitivity |
| SP | Specificity |
| T1 mapping | T1 Spin-Lattice Relaxation Time |
| TKV | Total Kidney Volume |
| U-Net | U-Shaped Neural Network |
| VGG16 | Visual Geometry Group With 16 Convolutional Layers |
| VGG19 | Visual Geometry Group With 19 Convolutional Layers |
|  |  |
