# Supplementary material for: Validation of automatically measured T1 map cortico-medullary difference (ΔT1) for eGFR and fibrosis assessment in allograft kidneys
Source: PLoS One. 2023 Feb 15;18(2):e0277277. doi: 10.1371/journal.pone.0277277 (PMC9931131; doi:10.1371/journal.pone.0277277)
Supplement: Attachment — Submitted filename: PLOSONE.docx [file pone.0277277.s004.docx]

Dear Editor

The article seems interesting in terms of content and preparation. The rate of chronic kidney failure is increasing with the increase of comorbid diseases (hypertension, diabetes, etc.) worldwide. As a renal replacement therapy, kidney transplantation is superior to other methods (hemodialysis, peritoneal dialysis).

The patient's primary disease, treatment compliance, immunosuppression, etc. are important in the success of the transplant. It is used in biopsy and imaging as well as laboratory in follow-ups. Scanning of the cortex medulla in MRI, as stated in the study, contributes to the diagnosis of interstitial fibrosis. In addition, confirmation of this in the laboratory and the contribution of image data in patients who develop rejection will help the clinician.

-One of the most important shortcomings of the study is the inability to compare clinical data with imaging. Is there a relationship between renal function, rejection and fibrosis with imaging?

The work will be more valuable if the specified features are highlighted.

Best regards
